# Basal level of ppGpp coordinates *Escherichia coli* cell heterogeneity and ampicillin resistance and persistence

**DOI:** 10.15698/mic2023.11.808

**Published:** 2023-10-25

**Authors:** Paulina Katarzyna Grucela, Yong Everett Zhang

**Affiliations:** 1 Department of Biology, University of Copenhagen, DK-2200 Copenhagen, Denmark.

**Keywords:** basal ppGpp, resistance, persistence, ampicillin, heterogeneity

## Abstract

The universal stringent response alarmone ppGpp (guanosine penta and tetra phosphates) plays a crucial role in various aspects of fundamental cell physiology (e.g., cell growth rate, cell size) and thus bacterial tolerance to and survival of external stresses, including antibiotics. Besides transient antibiotic tolerance (persistence), ppGpp was recently found to contribute to *E. coli* resistance to ampicillin. How ppGpp regulates both the persistence and resistance to antibiotics remains incompletely understood. In this study, we first clarified that the absence of ppGpp in *E. coli* (ppGpp^0^ strain) resulted in a decreased minimal inhibition concentration (MIC) value of ampicillin but, surprisingly, a higher persistence level to ampicillin during exponential growth in MOPS rich medium. High basal ppGpp levels, thus lower growth rate, did not produce high ampicillin persistence. Importantly, we found that the high ampicillin persistence of the ppGpp^0^ strain is not due to dormant overnight carry-over cells. Instead, the absence of ppGpp produced higher cell heterogeneity, propagating during the regrowth and the killing phases, leading to higher ampicillin persistence. Consistently, we isolated a suppressor mutation of the ppGpp^0^ strain that restored the standard MIC value of ampicillin and reduced its cell heterogeneity and the ampicillin persistence level concomitantly. Altogether, we discussed the fundamental role of basal level of ppGpp in regulating cell homogeneity and ampicillin persistence.

## INTRODUCTION

Bacteria constantly adjust their physiological and metabolic activities and even morphologies in response to unpredictable external environmental cues. Rapid growth rate under non-stressed conditions helps bacteria to compete for nutrients. In contrast, slow growth rate and non-growing dormancy state are essential for bacteria to survive harsh conditions, including starvation, predators, and attacks from the host cell immune systems. Therefore, it is imperative to understand how bacteria dynamically adjust their metabolism to better control them under unwanted conditions, e.g., during bacterial infection.

The stringent response alarmone ppGpp (guanosine penta and tetra phosphates) is a universal second messenger that plays a central role in bacterial physiological adaptations [1–5]. Initially discovered under *E. coli* amino acid starvation conditions [6], ppGpp was later found to be produced under various stress conditions, including the starvation of other nutrients (carbon [7], iron [8], fatty acid [9]). Consistently, ppGpp is essential for stress survival, bacterial virulence, and antibiotic tolerance, persistence, and resistance [1, 5, 10, 11]. The best-studied mechanism is how ppGpp helps *E. coli* to cope with amino acid starvation by RelA and “ribosome stalling” [12–15]. During amino acid starvation, the levels of uncharged tRNAs increase, which leads to the stalling of ribosomes on mRNA templates lacking cognate aminoacyl-tRNAs. This ribosome stalling leads to the activation of RelA, which senses the accumulation of uncharged tRNAs and responds by synthesizing ppGpp. ppGpp then binds to the RNA polymerase (RNAP) [16, 17] to reprogram global gene expressions, including decreased transcription of genes related to rapid growth and increased expression of genes related to stress response (e.g., amino acid biosynthesis genes), survival, and virulence [18–20]. Besides RNAP, ppGpp directly binds and affects the functions of numerous other proteins involved in *E. coli* translation and metabolism [21–24]. Altogether, ppGpp, via its numerous target proteins, promptly adjusts bacterial metabolism to adapt to various stresses.

Besides in adapting to stressful conditions, the most intriguing role of ppGpp is its connection with antibiotic persistence. Persistence is the phenomenon that a sub-population of isogenic bacterial cells survive the prolonged killing by lethal antibiotics [25, 26]. The persistent cells, i.e., persisters, contain no genetic mutation; after antibiotics are removed, persisters regenerate to produce a cell population that undergoes a similar antibiotic killing curve. These phenotypic persisters are thought to originate from noise and heterogeneity of genetic expressions [25, 27], can be induced by various stresses [11], and may contribute to recurrent and recalcitrant clinical bacterial infections. Moreover, many studies showed that high persistence facilitates bacterial survival of antibiotic killing and thus more likely leads to the gain of resistance mutations [28, 29], thereby multiple drug resistant superbugs.

In all studied bacteria, ppGpp is shown to be essential for persistence [11, 27, 30]. However, other factors, such as the toxin-antitoxin system [31–34], ATP [35, 36], and cell heterogeneity during the lag phase [37, 38], are also reported to affect persistence under various conditions. Intriguingly, a recent model proposed by the Groisman group suggests that slow and non-growing states dictate higher levels of persistence in *Salmonella* [39, 40], which seems promising to unify the diverse observations of persistence. Additionally, the same study found that under minimal medium starvation conditions the presence of ppGpp decreases, instead of increases, persistence [39]. The interpretation of these results lies on the fact that ppGpp supports active metabolism and cell growth, rendering them sensitive to antibiotics; while the absence of ppGpp did not allow cell growth, rendering them quiescent to antibiotic actions, leading to higher persistence levels. Notably, ppGpp is a well-established determinant of exponential cell growth rate in *E. coli* [41]. Artificially increasing ppGpp levels via mutations in SpoT, the other homolog protein of RelA in *E. coli*, has revealed an inverse relation between ppGpp levels and cell growth rate [41]. Therefore, one would expect a correlation between the level of ppGpp and persistence in exponentially growing non-stressed *E. coli* cells, which remains to be tested. Therefore, we first aimed to extend the persistence model proposed by Groisman *et al.* [40] to *E. coli*, by studying the persistence of strains producing varied basal levels of ppGpp and growing at different rates under non-stressed conditions.

Additionally, a new connection between ppGpp and antibiotic resistance was reported [42, 43]. A moderate expression of one L,D-transpeptidase YcbB, and simultaneous over-production of ppGpp made *E. coli* resistant to most β-lactam antibiotics, including ampicillin [43]. This was likely due to the replacement of the 4→3 peptidic cross-links catalyzed by PBPs (penicillin-binding proteins) by the 3→3 cross-links catalyzed by L,D transpeptidases. Since β-lactam antibiotics efficiently inhibit PBPs but not L,D-transpeptidase, using an alternative L,D transpeptidase provided an effective way to resist β-lactams. However, the mechanism of over produced ppGpp in this ampicillin resistance phenomenon remains still unclear. In this regard, previous transcriptomic studies showed that ppGpp influences expression of proteins involved in cell wall metabolism [18, 19]. A recent study also showed an inverse correlation between ppGpp levels and cell size of *E. coli* [44]. In accordance with this, the complete absence of ppGpp in the so-called ppGpp^0^ strain produces highly filamented *E. coli* cells [7]. These observations indicate a sophisticated relation between ppGpp, cell division, and ampicillin resistance, persistence.

In this study, we found that a basal level of ppGpp is essential for a high MIC value of ampicillin in *E. coli*. Unexpectedly, we found that exponentially growing cells of the ppGpp^0^ strain produced a higher level of ampicillin persistent cells than the wild type (wt) strain in MOPS rich medium. Further studies of ppGpp^0^ and its evolved suppressor strains, and strains with elevated basal ppGpp levels, allowed us to propose that the high cell heterogeneity of the ppGpp^0^ strain, represented by cell length difference, was responsible for its higher level of persistence towards ampicillin.

## RESULTS

### The absence of ppGpp reduces the ampicillin MIC value

We first tested the link between ppGpp and ampicillin by measuring the MIC values of the *E. coli* strains used, i.e., the wt MG1655 strain, the isogenic strain with the *relA* allele deleted (Δ*relA*), or with both the *relA* deleted and *spoT* replaced with a chloramphenicol resistance marker (Δ*relA* Δ*spoT207* strain [7], or the ppGpp^0^ strain). We chose to use the chemically defined rich MOPS medium supplemented with the twenty amino acids (MOPSr) [45] and 0.2 mM (instead of normally 1.32 mM) phosphate (for short, MOPSr-Lp). This choice was for several reasons. Firstly, the persistence in the LB complex medium varied dramatically due to batch variations of LB; in contrast, MOPSr-Lp contains explicit compositions of nutrients, enhancing data reproducibility. Second, we aimed to investigate the antibiotic responses of *E. coli* strains growing under non-stressed conditions. MOPSr-Lp provides all the necessary nutrients and trace elements for robust *E. coli* growth, facilitating our study. Lastly, the low phosphate concentration in MOPSr-Lp offered a robust method for labeling and measuring ppGpp levels with the radioactive isotope ^32^p-orthophosphate (see below).

**Figure 1:**
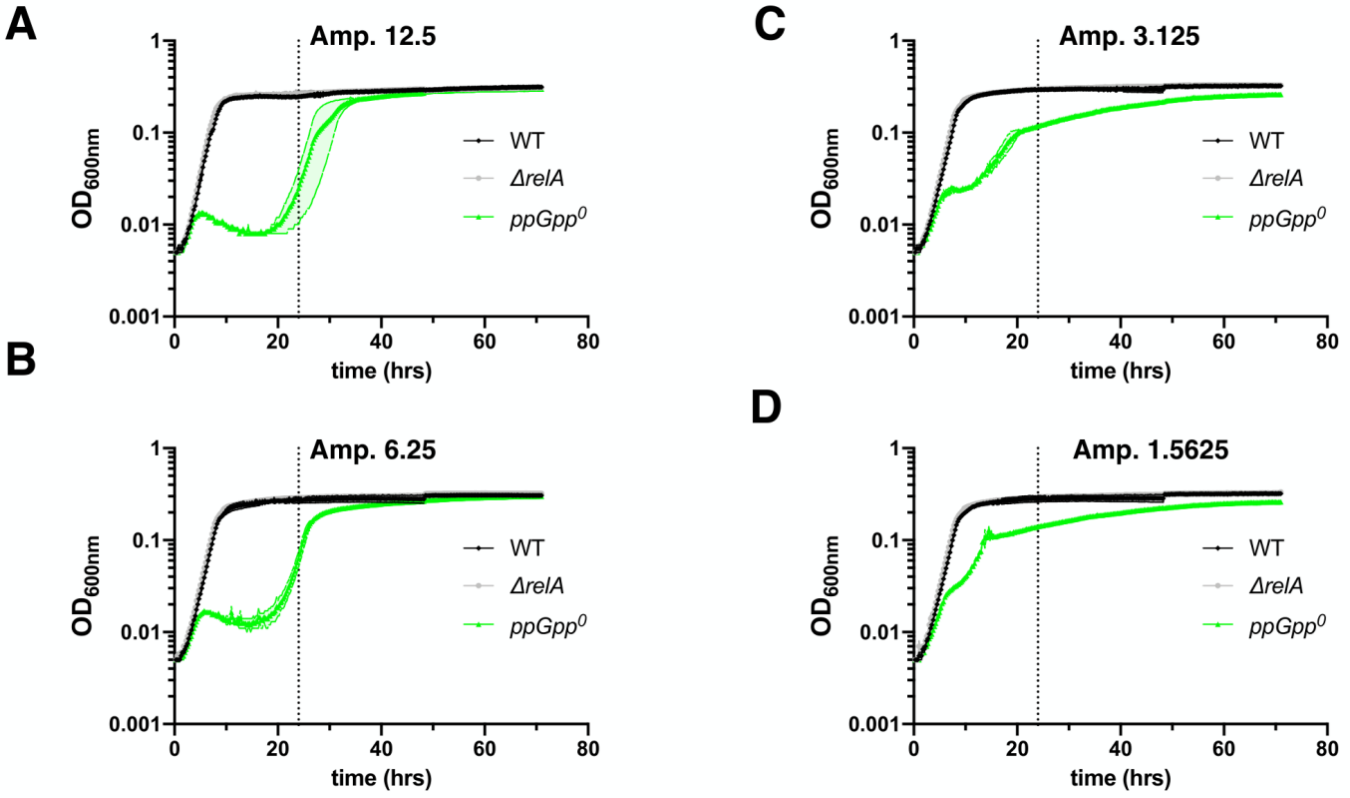
FIGURE 1: The absence of ppGpp reduces the MIC value of ampicillin in *E. coli*. (**A-D**) Growth curves of the three studied strains in MOPS rich medium supplemented with low (0.2 mM) phosphate (MOPSr-Lp) in the presence of two-fold serially diluted ampicillin (from 12.5 to 1.5625 μg/ml). Three biological replicates were performed and the averages and ranges were plotted. WT, *E. coli* MG1655; *ΔrelA, E. coli* MG1655 with the *relA* allele deleted; ppGpp^0^, *ΔrelA ΔspoT207*, with *spoT* replaced with a chloramphenicol resistance marker [7].

We found that up to 12.5 μg/ml ampicillin did not prevent or slow down the growth or the final yields of both wt and Δ*relA* strains in MOPSr-Lp (**Figure 1**). However, 12.5 μg/ml ampicillin completely inhibited the growth of the ppGpp^0^ strain for 24 hours, and two-fold serial diluted ampicillin from 12.5 to 1.5625 μg/ml slowed the growth of the ppGpp^0^ strain in a dose-dependent manner.

Since the ppGpp^0^ strain finally grew up after prolonged incubation (72 hrs) in the presence of inhibitory ampicillin, we asked if the ppGpp^0^ strain obtained any suppressing mutation(s). By re-growing the “evolved” cells in the presence of 6.25 or 3.125 μg/ml ampicillin, we found that they grew up readily within 24 hours and earlier than the original ppGpp^0^ strain (**Figure S1A**). This indicates that the ppGpp^0^ strain obtained some suppressing mutation(s), which allowed it to re-grow faster than the original strain and gain a higher MIC.

As controls, we tested two other antibiotics with different modes of action, i.e., gentamycin (targeting translation), and ofloxacin (targeting DNA replication). Despite the slowed growth of the ppGpp^0^ strain by both 0.125 μg/ml gentamycin and 0.03125 μg/ml ofloxacin (**Figure S1B-S1G**), the ppGpp^0^ strain did not obtain any resistance mutation after 72 hours of growth (**Figure S1H**), suggesting that the ppGpp^0^ strain has the same MIC values for both gentamycin and ofloxacin as the wt and Δ*relA* strains. Altogether, these data showed that the absence of ppGpp reduces the *E. coli* MIC value and makes it less resistant specifically to ampicillin.

### The exponential ppGpp^0^ cells are more persistent to ampicillin killing in MOPSr-Lp

**Figure 2:**
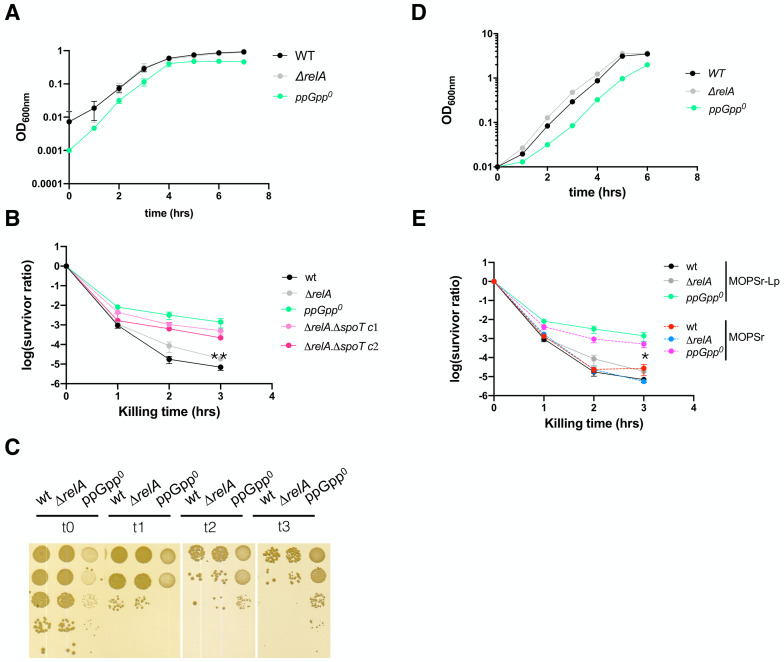
FIGURE 2: The exponential cells of ppGpp^0^ strain are more persistent to ampicillin killing than the wt and *ΔrelA* strains. **(A)** Growth dynamics of the wt, *ΔrelA* and ppGpp^0^ strains in MOPSr-Lp. Four biological replicates were performed, and the averages and SDs are shown. **(B)** The ampicillin (250 μg/ml) killing curves of the exponentially growing wt, *ΔrelA* and ppGpp^0^ strains in MOPSr-Lp. Six biological replicates were performed, and the averages and SDs are shown. *ΔrelA ΔspoT c1, c2*, two separate colonies of the in-frame deletion mutants of both *relA* and *spoT* genes. **, p<0.01, student t-test, between *ΔrelA* and ppGpp^0^. **(C)** One representative plate of the surviving cells after ampicillin killing in panel B. **(D)** Growth dynamics of the wt, *ΔrelA* and ppGpp^0^ strains in MOPSr medium with the standard amount of phosphate. Two biological replicates were performed, and the averages and SDs are shown. **(E)** The ampicillin (250 μg/ml) killing curves of the exponentially growing wt, *ΔrelA* and ppGpp^0^ strains in MOPSr. Six biological replicates (MOPSr) were performed, and the averages and SDs are shown. For comparison, the same killing curves of the three strains from (B) were included.

By definition, a lower ampicillin MIC of the ppGpp^0^ strain indicates that the cell population was not killed but their growth was inhibited by ampicillin. The ppGpp^0^ cells survived the prolonged ampicillin treatment to eventually evolve out with resistance mutation(s) (**Figure S1A**), suggesting that the ppGpp^0^ cells tolerated the sub-MIC ampicillin well. We thus tested if the ppGpp^0^ strain tolerates a lethal concentration, i.e., 250 μg/ml, ca. 10-20 x MIC of ampicillin, in a persistence assay. To avoid other confounding factors (e.g., stress), we tested exponentially growing cells. For this, 16-hr overnight cultures of the wt, Δ*relA,* and the ppGpp^0^ strains were inoculated into fresh pre-warmed MOPSr-Lp medium and allowed to grow for 2.5 hrs to the early exponential phase (**Figure 2A**). Ampicillin (250 μg/ml) was then added to kill the cells and the killing kinetics were determined hourly for 3-5 hrs. Surprisingly, the ppGpp^0^ strain had significantly more persister cells than Δ*relA* and wt strains (**Figure 2B**) and the differences are highly reproducible (six biological replicates, t-test, p=0.011 and p=0.0052 for 3 hr killing time point*,* respectively). Of note, the colonies of the surviving ppGpp^0^ cells were smaller, indicative of slower growth and recovery on LB medium (**Figure 2C**). These small colonies did not contain resistance mutations since a second killing of these colonies in fresh MOPSr-Lp medium produced the same curves as the original ppGpp^0^ strain (data not shown).

To further confirm this unexpected phenomenon of the ppGpp^0^ strain, we re-constructed an in-frame clean deletion mutant of *spoT*, i.e., Δ*relA* Δ*spoT*, using the scarless method (see Material and Method) [46]. We tested two separate colonies of Δ*relA* Δ*spoT* and found similar killing curves of ampicillin as for the ppGpp^0^ strain (**Figure 2B**). In conclusion, both ppGpp^0^ strains were more persistent to ampicillin than the Δ*relA* and wt *E. coli* strains. We further performed persistence assays for two other antibiotics, i.e., gentamycin and ofloxacin (**Figure S2**). For gentamycin, we did not observe a significant difference in persistence among the strains. For ofloxacin, we observed a slightly higher level of persistence (<10-fold) in the ppGpp^0^ strain compared to the Δ*relA* strain. This finding suggests that the high persistence and low MIC value observed in the ppGpp^0^ strain are specific to ampicillin.

### High ampicillin persistence of ppGpp^0^ strain holds in standard MOPSr medium

The lower MIC value and higher persistence of the ppGpp^0^ strains to ampicillin were puzzling. The low phosphate used in the MOPSr-Lp medium may have caused this phenomenon. To test this, the growth dynamics (**Figure 2D**) and killing assay (**Figure 2E**) were confirmed in MOPSr supplemented with the standard 1.32 mM phosphate. The persistence level of the wt and Δ*relA* strains did not change significantly as compared to those in MOPSr-Lp medium. Despite a small drop of persistence level, the ppGpp^0^ strain had still significantly more persistent cells than wt and Δ*relA* (six biological replicates, t-test, p=0.0258 and p=0.0207 for 3 hr killing time point*,* respectively). This argues that the high persistence of the ppGpp^0^ strain cannot be attributed to the low phosphate in MOPSr-Lp.

### A lower growth rate did not dictate higher ampicillin persistence in MOPSr-Lp

**Figure 3:**
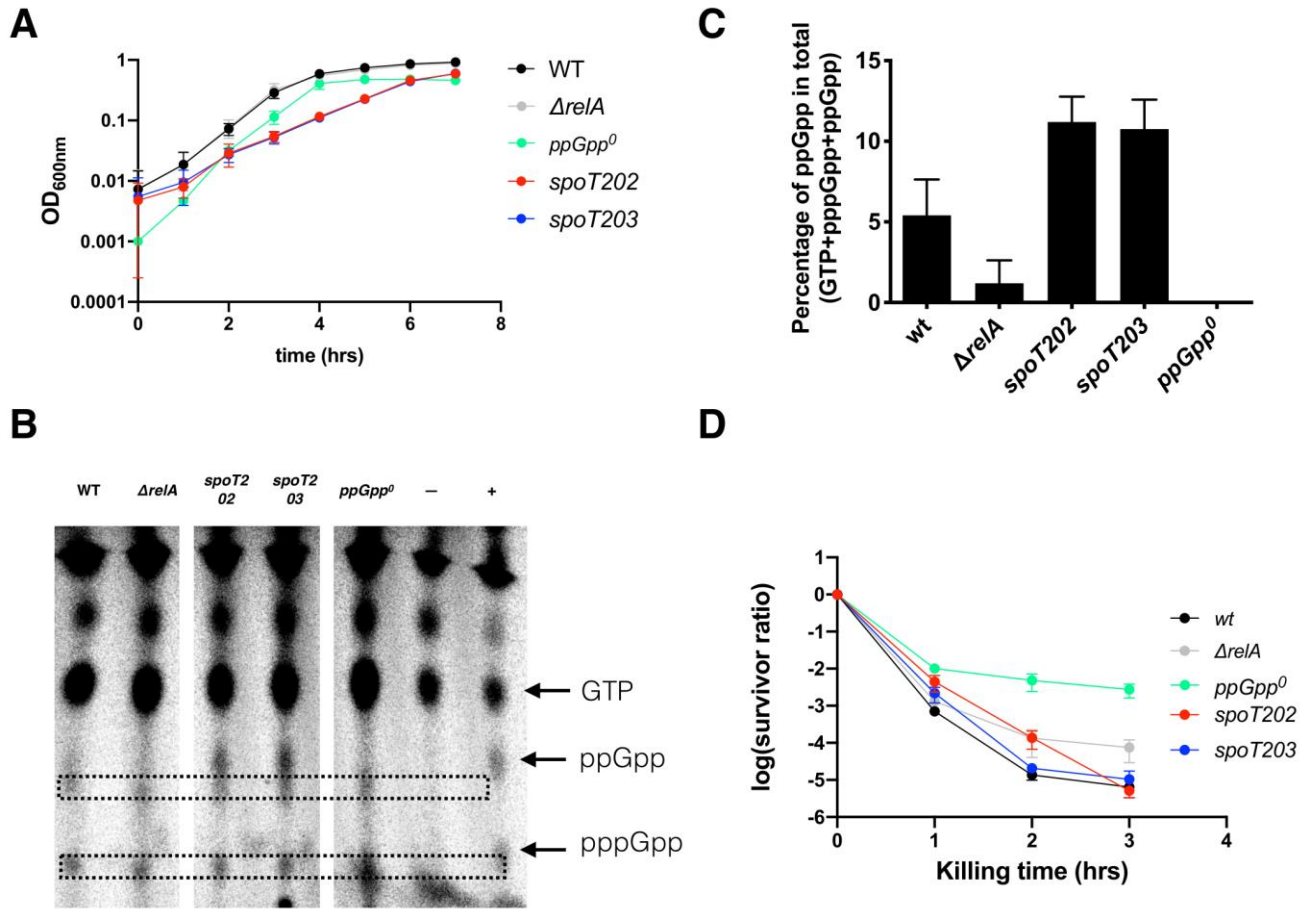
FIGURE 3: High basal ppGpp and thus low growth rate did not produce more ampicillin persistent cells. **(A)** Growth dynamics of the *spoT202* and *spoT203 strains*. For comparison, the growth curves of the wt, *ΔrelA* and ppGpp^0^ strains from Figure 2A were included. Four biological replicates were performed, and the averages and SDs are shown. *spoT202, ΔrelA spoT* (T78I) [47]; and *spoT203, ΔrelA spoT* (R140C) [47]. **(B)** Thin layer chromatography analysis of the intracellular ppGpp levels in the five exponentially growing strains as labeled with ^32^p-orthophosphate in the MOPSr-Lp medium. GTP, ppGpp and pppGpp are indicated. Unknown molecules nearby the ppGpp are framed in broken-line rectangles. **(C)** Quantification of the ppGpp levels as in panel (B). Two biological replicates were performed, and the average and SD are shown. **(D)** The ampicillin (250 μg/ml) killing curves of the studied strains. Three biological replicates of both *spoT202* and *spoT203* were performed, and the averages and SDs are shown. For comparison, the same killing curves of the three strains from (Figure 2B) were included.

The ppGpp^0^ strain has a slightly lower growth rate (doubling time 0.54 ± 0.02 hr) than the wt and *relA* strains (0.48 ± 0.02 vs. 0.48 ± 0.03 hr, respectively, **Figure 3A**), which might cause the higher persistence to ampicillin according to the proposed model [39, 40]. Additionally, the ppGpp level dictates the *E. coli* growth rate [41]. To test if a higher basal ppGpp level, thus a lower growth rate, leads to higher persistence, we employed strains of *E. coli* K-12 carrying point mutations in *spoT* known to produce higher basal levels of ppGpp. These strains included the SpoT202 (T78I) [47] and SpoT203 (R140C) [47]. Isogenic MG1655 strains carrying the mutations were reported to produce 7- and 4-times increased levels of ppGpp as compared to wt. SpoT is essential when RelA is present in MG1655, and *relA* is dispensable for cell growth in rich MOPSr medium with twenty amino acids. To avoid potential influence from the residual RelA activity and simplify the genetic engineering of *spoT* on the chromosome, we introduced these SpoT mutations in the endogenous *spoT* allele in the Δ*relA* deletion strain of MG1655 by using the scar-less method.

We found that wt MG1655 re-grew from a 16-hr overnight culture as early as the strains Δ*relA*, followed by the ppGpp^0^ strain, then *spoT202* and *spoT203* (**Figure 3A)**. These lead to varied doubling times of the strains, with *spoT202 and spoT203* exhibiting a growth rate that was half of the wt, Δ*relA* and the ppGpp^0^ strains (doubling time 1.02 ± 0.05 and 0.99 ± 0.08 hr, respectively). These data confirm that 1) RelA is not required for the growth of *E. coli* in MOPSr-Lp; 2) that the expected high level of ppGpp in *spoT202 and spoT203* reduced their growth rates significantly [47]. To corroborate, we measured the intracellular ppGpp with the radioactive ^32^p-orthophosphate (**Figure 3B)** [21]. As expected, the exponentially growing cells of both *spoT202* and *spoT203* strains produced higher ppGpp levels than the wt strain, which is higher than both Δ*relA* and the ppGpp^0^ strains with very low, if any, basal levels of ppGpp (**Figure 3C)**.

With the *spoT* mutant strains constructed and their ppGpp levels confirmed, we then sought to use them to study the effect of higher basal ppGpp levels and, thus slower growth rate on ampicillin persistence. Surprisingly we found that both the *spoT202* and *spoT203* strains were rapidly killed by ampicillin, and after three hours, both had similar persistence levels to that of the wt strain (**Figure 3D**). These data demonstrated that higher basal levels of ppGpp and thus lower growth rates do not lead to higher ampicillin persistence in exponentially growing cells in MOPSr-Lp.

### Overnight carry-over cells did not fully account for the high persistence of ppGpp^0^ strain

**Figure 4:**
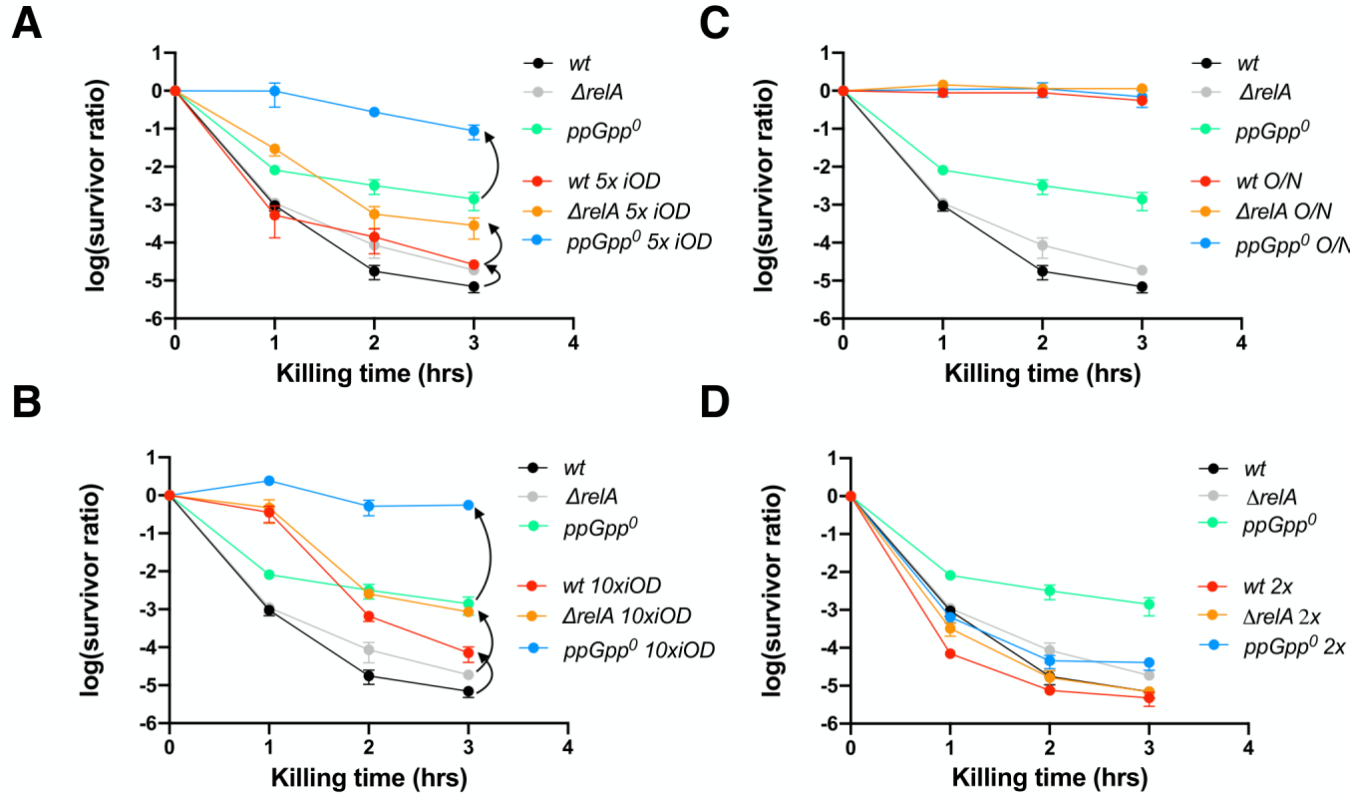
FIGURE 4: Overnight dormant cells did not fully account for the ampicillin persistent cells of ppGpp^0^ strain. (**A, B**) The ampicillin (250 μg/ml) killing curves of the exponentially growing wt, *ΔrelA* and ppGpp^0^ strains. **(A)** 5x iOD, five times more overnight cells (initial OD_600nm_ = 0.05) were inoculated into fresh MOPSr-Lp medium and grew to the exponential phase before being killed with ampicillin. **(B)** 10x iOD, ten times more overnight cells (initial OD_600nm_ = 0.1) were inoculated into fresh MOPSr-Lp medium and grew to the exponential phase before being killed with ampicillin. Two biological replicates of both 5x iOD and 10x iOD were performed, and the averages and SDs are shown. **(C)** The ampicillin (250 μg/ml) killing curves of the wt, *ΔrelA* and ppGpp^0^ cells directly out of the 16-hour overnight cultures (indicated by O/N). Two biological replicates were performed, and the averages and SDs are shown. **(D)** The ampicillin (250 μg/ml) killing curves of the wt, *ΔrelA* and ppGpp^0^ cells after subculturing twice in fresh MOPSr-Lp medium (indicated by 2x). After 3 hours of first subculturing in fresh MOPSr-Lp medium, cells were subcultured again from OD_600nm_ 0.01 for 2.5 hrs before being subjected to killing. Two biological replicates were performed, and the averages and SDs are shown. For comparison, the same killing curves of the three strains from (Figure 2B) were included throughout.

Both wt MG1655 and Δ*relA* re-grew rapidly and earlier than the ppGpp^0^ strain in MOPSr-Lp (**Figure 3A).** A more extended lag phase is known to contain a subpopulation of cells in a dormant state that avoids the killing by antibiotics. If true, one would expect to see proportionally more persistent cells when more overnight cells are inoculated. To test this, we increased the inoculum by five or ten times and tested the persistence again (**Figure S3**). We found that the persistent cells of the ppGpp^0^ strain increased by ca. 100 x and 1000 x fold, respectively, when the inoculum increased by 5 x and 10 x fold (**Figure 4A, 4B**). On the other hand, the persistent cells of both wt and Δ*relA* strains increased less dramatically (10-100 x fold). As controls, the overnight cells were completely resistant to the killing, consistent with the fact that ampicillin kills actively growing and dividing cells (**Figure 4C**). Therefore, despite that the increased persistent cells of all three strains can be partially from the overnight dormant cells, another factor contributes significantly more to ampicillin persistence during regrowth, and such a persistence “amplification” effect is more pronounced in the ppGpp^0^ strain than in wt and Δ*relA* strains.

We hypothesized that the high ampicillin persistence of the ppGpp^0^ strain could be caused by a higher cell heterogeneity during regrowth. Subculturing cells one more time should reduce both dormant cells, lag time, and cell heterogeneity and, thus the persistence level. To test this, we subcultured the strains first for 3 hrs from OD_600nm_ 0.01, then subcultured the cells again from OD_600nm_ 0.01 for 2.5 hrs before subjecting them to killing. We observed a drop in persistence of all three strains*,* but the ppGpp^0^ strain still produced ten times more persistent cells than the wt and Δ*relA* strains (**Figure 4D**, 3 hr time point). This result indicates that the exponentially growing ppGpp^0^ cells produce high cell heterogeneity in MOPSr-Lp, leading to high persistence to ampicillin.

### High cell heterogeneity of the ppGpp^0^ strain during regrowth and ampicillin killing

**Figure 5:**
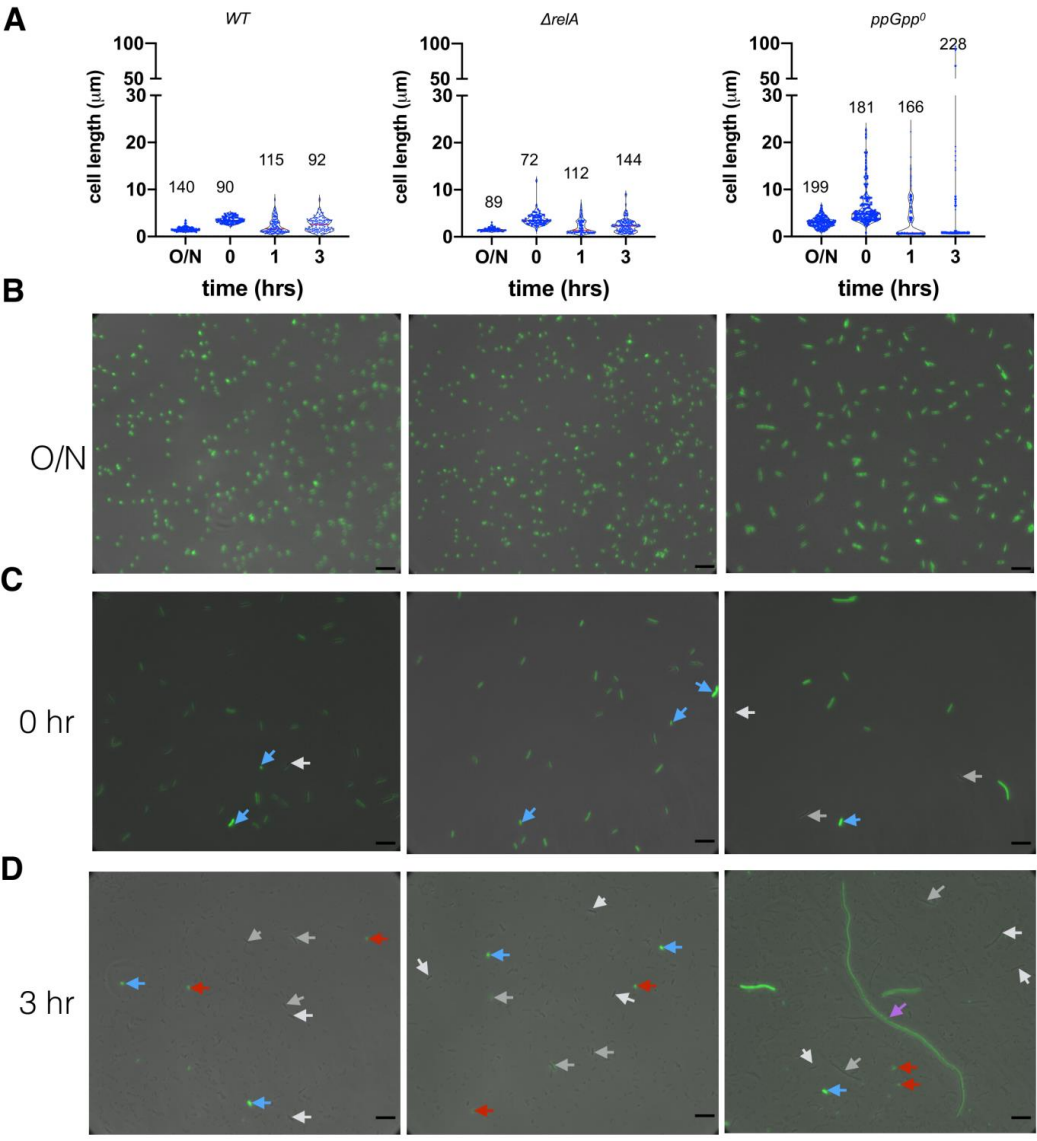
FIGURE 5: Heterogeneity of cells before and during ampicillin killing. **(A)** Violin plot of the cell length (µm) of the wt, *ΔrelA* and ppGpp^0^ cells under different conditions. O/N, 16-hr overnight cells in MOPSr-Lp medium; time point t0, 2.5 hr regrowth in fresh MOPSr-Lp medium from OD_600nm_ 0.01; time points t1 and t3, cells after killing by ampicillin for 1 and 3 hrs respectively. The numbers of analyzed cells are indicated on top of the plots. (**B, C, D**) Representative microscopic pictures of cells at different time points. The phase contrast pictures are overlaid with the GFP fluorescence images. White and grey arrows, cells with no or low green fluorescence; blue arrow, potential overnight carry-over cells with strong green fluorescence; red arrow, dying round mini cells; magenta arrow, elongated cells of ppGpp^0^. Scale bars are 6 µm.

The above data suggest that the heterogeneity of the ppGpp^0^ strain leads to more persistent cells to ampicillin during regrowth in the rich MOPS medium. To corroborate this, we constructed the p7 GFP gene [48] under the pBAD promoter of pBAD33 and transformed the plasmid into the three strains. Arabinose was added into the pre-cultures in MOPSr-Lp to express the p7 GFP. The arabinose was then washed away to stop further expression of p7 GFP. These cells were used to inoculate fresh MOPSr-Lp, and similar growth dynamics and ampicillin killing curves were observed, indicative of no major effect from p7GFP expression (data not shown). The exponential phase cells of these strains were subjected to ampicillin killing, and cells were inspected at different time points under a fluorescence microscope (**Figure 5**).

For stationary phase cells, we observed *E. coli* cells with characteristic short rods for both wt and Δ*relA* strains (cell length 1.57 ± 0.46 µm, N=140, and 1.55 ± 0.37 µm, N=89, respectively; student t-test p=0.733). The ppGpp^0^ cells, however, were significantly longer (3.09 ± 1.09 µm, N=199, student t-test p <0.0001 between ppGpp^0^ and Δ*relA*), consistent with previous report [7]. Further, the ppGpp^0^ cells showed a higher heterogeneity in terms of cell length (**Figure 5A, 5B;** SD/average ratio 0.35 of ppGpp^0^ vs. 0.24 of Δ*relA*) [49], suggesting a role of basal ppGpp in regulating cell length and size homogeneity. All cells of the three strains showed a strong green fluorescence of p7 GFP (**Figure 5B**). Upon regrowth to the exponential phase (time point 0, **Figure 5C**), both wt and Δ*relA* cells elongate to 3.56 ± 0.69 µm and 3.74 ± 1.4 µm with good uniformity (SD/average ratio 0.19 and 0.37), while a more significant cell length heterogeneity of ppGpp^0^ was observed (6.15 ± 3.9 µm, SD/average ratio 0.63) as in [7]. Some ppGpp^0^ cells are 12-22 µm long, which was not observed for the wt and Δ*relA* strains. Note that the varied dim green fluorescence intensity in cells indicates a dilution of the stable p7 GFP through cell divisions (white and grey arrows, **Figure 5C**). We also noticed potential overnight carry-over dormant cells with strong fluorescence intensity (blue arrows, **Figure 5C**).

The 3-hr killing by ampicillin led to an even higher cell-length heterogeneity of all strains, because of the production of round, highly fluorescent, thus likely dying, mini cells (average diameter ca. 0.65 µm; **Figure 5D**, red arrows). The wt and Δ*relA* cells did not elongate during ampicillin killing, and fluorescence was dim for most of the surviving cells (**Figure 5A**, **5D**, white and grey arrows), suggesting they are offspring cells after several rounds of cell division. The ppGpp^0^ strain also produced dim surviving cells with intermediate length (ca. 15 µm; white and grey arrows). Remarkably, some humongous, elongated cells (magenta arrow, **Figure 5D**) of ppGpp^0^ were observed, which were ca. 90 µm long, thus 4-15 times longer than normal time zero ppGpp^0^ cells (**Figure 5C**). These cells emitted weak green fluorescence as compared to the bright dormant cells (blue arrow), indicating that they were overnight cells growing out of the stationary phase. However, their significantly increased cell length indicates that these cells were growing in MOPSr-Lp despite the presence of a lethal concentration of ampicillin. Although dormant bright cells (blue arrows) were found for all three strains, we believe that the dim and elongated growing cells of ppGpp^0^ likely contributed to its significantly more persistent cells to ampicillin.

### The ppGpp^0^ suppressing mutants with rescued ampicillin MIC become less heterogeneous and persistent

**Figure 6:**
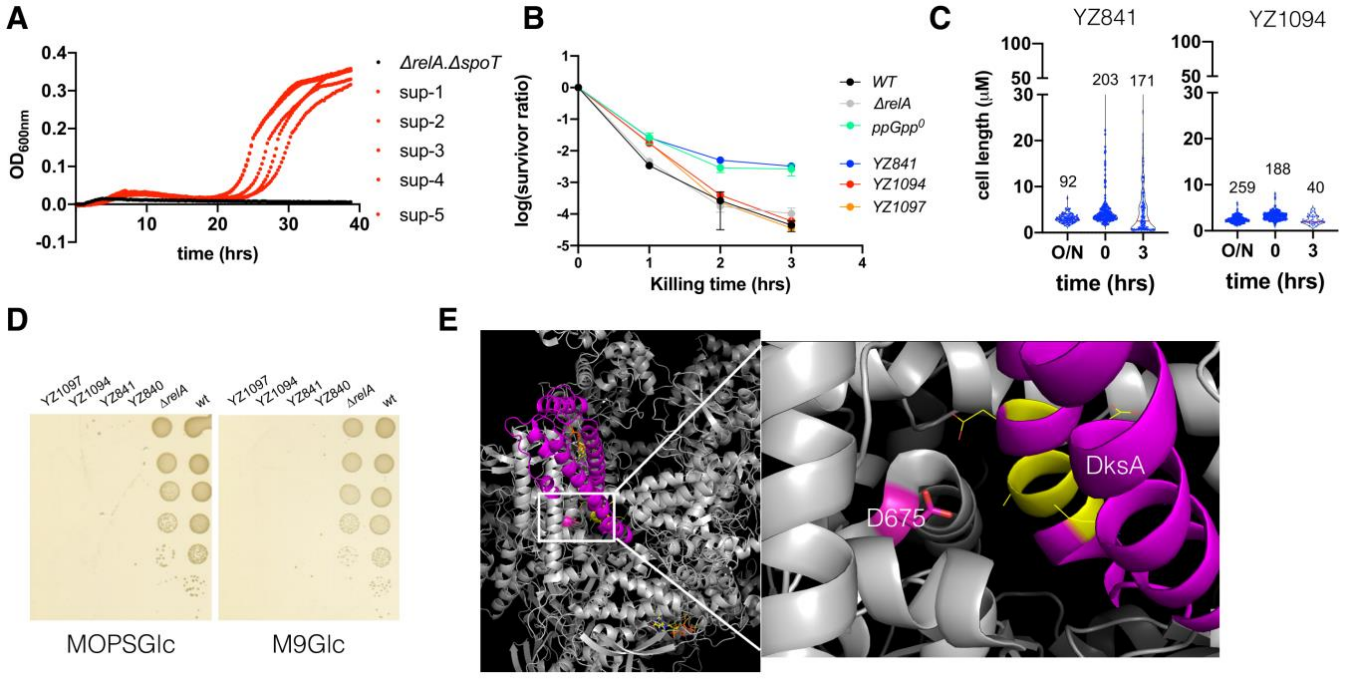
FIGURE 6: The ppGpp^0^ suppressing mutants rescued the ampicillin MIC and are less heterogeneous and persistent. **(A)** Growth curves of the parental *ΔrelA ΔspoT* strain and the five suppressor strains in the presence of 12.5 μg/ml ampicillin. **(B)** The ampicillin (250 μg/ml) killing curves of the parental *ΔrelA ΔspoT* strain (YZ841) and the two suppressor strains (YZ1094, YZ1097). Three biological replicates were performed, and the average and SEM were presented. For comparison, the same killing curves of the three strains from (Figure 2B) were included. **(C)** Box plot of the cell length (µm) of the parental *ΔrelA ΔspoT* strain and the suppressor strain YZ1094 cells during regrowth and ampicillin killing. The numbers of analyzed cells are indicated above the plots. **(D)** Growth of the parental *ΔrelA ΔspoT(*YZ840/YZ841*)* and the suppressor strains (YZ1094/YZ1097) on MOPS (left) and M9 (right) minimal media agar plate supplemented with glucose 0.2% g/ml (MOPSGlc and M9Glc). Pictures were taken after 24 hrs growth at 37°C. **(E)** 3D position of the suppressing mutation D675A on RpoB, in a complex structure of *E. coli* RNAP (grey), DksA (magenta), and two molecules of ppGpp (yellow stick model) (PDB 5VSW) [50]). D675 is shown in a stick model (magenta). To the right side is a zoom-in of the framed region, with DksA residues directly facing D675 shown in yellow and line model.

The ppGpp^0^ strain has lower ampicillin MIC (**Figure 1**), and the suppressing mutants (hereinafter called ppGpp^0^-AS) frequently occurred (**Figure S1A**). We isolated several ppGpp^0^-AS strains after 72 hrs of growth of the strain Δ*relA* Δ*spoT* (YZ841) in the presence of ampicillin (12.5 μg/ml). We found that the tested five ppGpp^0^-AS strains all grew up in the presence of ampicillin and thus restored the ampicillin MIC to the standard value of wt and Δ*relA* strains (**Figure 6A**), indicating stable mutation(s) were present in these strains. We also found that these ppGpp^0^-AS strains (YZ1094, YZ1097) became less persistent to ampicillin and behaved like the wt and Δ*relA* strains (**Figure 6B**). Moreover, the suppressing strains showed rescued heterogeneity in cell length during both regrowth and ampicillin killing as compared to the parental strain Δ*relA* Δ*spoT* (YZ841) (**Figure 6C**).

It is known that the ppGpp^0^ strain frequently obtains mutations during the stringent response. To check if the ppGpp^0^-AS strains contain a stringent mutation, we tested their growth on MOPS and M9 minimal media supplemented with glucose 0.2% (g/ml) but without amino acids. **Figure 6D** shows that, like the parental strains, none of the ppGpp^0^-AS strains grew on either medium, while the positive control strains wt and Δ*relA* did. These data suggest that the suppressing mutations encoded in these ppGpp^0^-AS strains are not stringent. To identify the mutation(s), we first amplified the *rpoB* gene of the two ppGpp^0^-AS strains since *rpoB* is a mutation hot spot. Sanger sequencing revealed that an A2024C mutation occurred on both YZ1094 and YZ1097 strains, leading to a D675A amino acid change. D675 is a highly conserved residue and confronts the backbone of DksA residue Alanine77 (PDB 5VSW, [50]; **Figure 6E**). Further whole genome sequencing revealed no other mutation besides A2024C, suggesting that D675A was responsible for restoring the typical MIC of ampicillin, homogenous cell length, and reduced persistence to ampicillin of the ppGpp^0^ strain.

## DISCUSSION

In this study, we found that the absence of ppGpp reduced the MIC value of ampicillin in *E. coli* and, surprisingly, that exponentially growing cells of the ppGpp^0^ strain are more persistent to ampicillin than the wt and Δ*relA* strains. Further experiments showed that a higher basal level of ppGpp and thus lower growth rate did not lead to high ampicillin persistence under non-stressed conditions (**Figure 3**). Instead, we found that the high persistence levels to ampicillin cannot be (solely) attributed to slow or absence of cell growth, or overnight carry-over cells, but rather was due to a high cell heterogeneity, represented by cell length/size (**Figures 4, 5**). Many studies showed that high antibiotic persistence facilitates bacterial gain of resistance mutations [28, 29]. Significantly, we readily isolated a suppressor mutation of ppGpp^0^ strains, which concomitantly restored the lower MIC value, and reduced its cell homogeneity and the persistence level to ampicillin (**Figure 6**), strongly arguing for a critical role of basal levels of ppGpp in regulating cell heterogeneity and ampicillin persistence and resistance.

The lower ampicillin MIC of the ppGpp^0^ strain is consistent with other reports; however, the high ampicillin persistence of exponentially growing ppGpp^0^ strain appears to contradict with many other studies. We suspect that several reasons may account for this discrepancy. 1) We used MOPSr chemically defined rich medium, while many other studies used LB complex medium. 2) We made and used the *E. coli* K-12 MG1655 strain and its isogenic strains with in-frame clean deletions or mutations of *relA* and *spoT* in the endogenous chromosomal loci. This minimizes unnecessary heterogeneity derived from e.g., gene dosage difference from plasmid vectors, residual antibiotic marker of the e.g., KEIO collection strains [51]. 3) We studied the exponential phase cells, which are not expected to experience significant stress except for the varying levels of basal ppGpp (**Figure 3**). This allowed us to directly test the hypothesis that basal ppGpp levels dictate the persistence level. Indeed, the persister cells we studied can be viewed as the spontaneous (type II) persisters generated by non-stressed cells, which have been rarely studied [25], whereas, most other persistence studies involve cells experiencing some stress conditions that generate type I persisters [11]. These stresses include, for instance, nutrient starvation, low pH, low oxygen, and host cell immune system. Under these stressful conditions, ppGpp firstly is essential for transcribing genes responsible for bacterial coping with these stresses [5]. The absence of ppGpp thus predisposed bacteria to these stresses, further confounding the study of its role on persistence. 4) Lastly, the high ampicillin persistence phenomenon was observed before in exponential phase cells of *E. coli* ppGpp^0^ strain [11]. Furthermore, we tested two different ppGpp^0^ strains and six biological replicates produced highly consistent results (**Figure 2**), suggesting that this phenomenon was authentic.

How to interpret the high ampicillin persistence of the ppGpp^0^ strain growing under non-stressed conditions? The ppGpp^0^ strain of *E. coli* was known many years ago to form long filamentous cells [7]. Consistently, we found increasing heterogeneity of exponential cells from the wt, Δ*relA* to the ppGpp^0^ strains (**Figure 5A**). This agrees with the gradually increased cell size (and heterogeneity) of *E. coli* when the level of ppGpp was tuned down from near stringent to sub-basal levels [44]. In accordance, we observed rapid killing by ampicillin of the wt strain, intermediate killing of Δ*relA* and the slowest killing of the ppGpp^0^ strain (**Figure 2B**). Furthermore, the strains *spoT202* and *spoT203* contain higher levels of basal ppGpp but they are less persistent to ampicillin (**Figure 3**). We propose that both mutants with higher basal levels of ppGpp exhibit greater control over cell size heterogeneity that is comparable to wt cells, but is higher than the Δ*relA* strain, and finally the ppGpp^0^ strain. Conversely, wt cells exhibited a persistence level similar to that of both mutants, while the Δ*relA* strain displayed higher persistence, with the highest level being observed in the ppGpp^0^ strain. In total, it appears that under non-stressed exponentially growing conditions, basal levels of ppGpp are inversely corelated with cell heterogeneity and ampicillin persistence.

Despite the correlation, the underlying mechanism of how ppGpp regulates cell size heterogeneity remained elusive. In this study, we obtained random suppressors of the ppGpp^0^ cells following exposure to sub-MIC levels of ampicillin (**Figure 6**). These suppressors not only restored a normal ampicillin MIC value but also led to a simultaneous reduction in both cell heterogeneity and ampicillin persistence. These observations strongly suggest a pivotal role of basal ppGpp in orchestrating cell growth, division, and, consequently, cell size heterogeneity and ampicillin persistence. To explore this correlation further, we attempted to analyze the gene expression differences among ampicillin-persistent cells from wt, Δ*relA*, and ppGpp^0^ strains using bulk RNA sequencing. However, due to the significant variability in the samples, likely stemming from the high heterogeneity of persister cells, we were unable to draw definitive conclusions from these data (not shown). Instead, we believe single persister cell sorting and single-cell RNA sequencing may hold the potential. Despite this, many similar analyses were performed in *E. coli* cells under stringent conditions, thus providing a picture of how stringent, high level of ppGpp controls the transcription of individual genes. For example, Traxler *et al.* probed transcriptomic differences between wt MG1655 and a ppGpp^0^ mutant during isoleucine starvation [19]. Fifteen genes involved in cell division significantly differed between the two strains, with nine up-regulated (i.e., *yibP*/*envC, tig, gidA, ftsX, ftsP, cedA, obgE, nlpI, rrmJ*) and six down-regulated (i.e., *xerC, tolC, yjbQ, bolA, fic, dacC*/*pbp6*) in the ppGpp^0^ strain. Importantly, Uehara *et al.* showed that four LytM domain proteins, including EnvC, are essential to activate amidases for segregating daughter cells [52]. Indeed, the absence of the four LytM domain proteins (Δ*envC* Δ*nlpD* Δ*ygeR* Δ*yebA*) made *E. coli* cells elongate and less sensitive to ampicillin killing [52]. Besides, endopeptidases such as MepS and MepM are essential for 3→3 based peptidoglycan network expansion during cell growth [42]. Moreover, the outer membrane lipoprotein NlpI potentially interacts with murein endopeptidases PBP4/1, PBP7, MepS, and MepM [53]. Altogether, given the effect of ppGpp on gene expression of multiple cell wall related enzymes, it is conceivable that some dysregulation of cell wall growth and cell division occurs in the ppGpp^0^ strain, thereby leading to the enhanced cell size heterogeneity.

How does then the higher cell size heterogeneity contribute to high ampicillin persistence of ppGpp^0^ cells? One possible explanation is that ppGpp^0^ cells are predisposed to hyper-respond to ampicillin given the proposed dysregulated gene expression pattern [19] and thereby increase the persistence level [40]. However, we observed actively growing cells of ppGpp^0^ during the ampicillin-killing phase (**Figure 5D**), indicating that it was not cell dormancy which prevented their killing by ampicillin. Instead, the morphological change of ppGpp^0^ cells during both regrowth and ampicillin killing phase suggest dramatic physiological changes that either pre-exist or are exaggerated further by ampicillin treatment, or more likely, both. Cell lysis rates induced by β-lactams correlate with bacterial growth rate [54] and, importantly, cell lysis requires a functional cell division machinery, the divisome [55]. The filamented cells of the ppGpp^0^ strain indicate a malfunctional divisome, which may somehow link to its higher persistence, similar as the quadruple mutant of LysM domain proteins reported in [52]. Further, *E. coli* cells killed by ampicillin are known to elongate during the process, generating a characteristic cell morphology that can be used to distinguish modes of action of different antibiotics [56]. Using these experimental data, recent mathematical modeling took a step further and suggested that a lower surface-to-volume (S/V) ratio of *E. coli* growing under non-stressed condition are beneficial for antibiotic resistance [57]. In this scenario, elongated cells have a lower S/V ratio, thus a lower influx rate of antibiotics, while rich medium supports fast growth, thus producing ample antibiotic targets and a fast dilution of effective antibiotics. ppGpp^0^ cells are naturally longer during regrowth in non-stressed condition (**Figure 5C**); meanwhile, rich MOPS medium supports active growth of ppGpp^0^ cells even during ampicillin killing, as estimated from the highly elongated cells (**Figure 5D**). Altogether, it is conceivable that the ppGpp^0^ cells have malfunctional divisomes that lead to high cell heterogeneity, which potentially serve as a bet-hedge strategy to avoid ampicillin killing. Despite these plausible connections, a mechanistic study is highly warranted to explicitly uncover the underlying mechanism of ppGpp in regulating cell heterogeneity and high ampicillin persistence and resistance.

## MATERIALS AND METHODS

### Bacterial strains, growth, media, and antibiotics

The constructed strains and primers used in this project are listed in Table S1 and S2, respectively. The *E. coli* K-12 MG1655 is the wild type strain used. Lysogeny broth (LB, containing 10 g tryptone (Oxoid), 5 g yeast extract (Oxoid), and 10 g NaCl (SIGMA) per liter of distilled water) was the primary medium used for *E. coli* growth and cloning. The defined MOPS rich medium (MOPSr) is the same as in [45]. The defined MOPS rich medium with low phosphate is the same as MOPSr, except that 0.2 mM, instead of 1.32 mM, K_2_HPO_4_ is supplemented. The MOPSGlc medium was the same as MOPSr, except that no amino acid was supplemented. M9 minimal medium (M9Glc) contains Na_2_HPO_4_ 48 mM, KH_2_PO_4_ 22 mM, pH 7.4, NaCl 8.6 mM, NH_4_Cl 18.7 mM, MgSO_4_ 1 mM, CaCl_2_ 0.1 mM, thiamine 1 μg/ml, glucose 0.2% g/ml. When applicable, 1.5% g/ml agar is added to make agar plates. Chloramphenicol 25 μg/ml, ampicillin 100 μg/ml, and anhydrotetracycline were used for the scarless cloning process.

### Scarless chromosomal mutagenesis of spoT

To introduce a point mutation in the *spoT* gene on the chromosome, the scar-less mutagenesis method reported in [46] was used. Briefly, primers PYZ395/396 were used to amplify the SceI-Cam fragment from the pWRG100 plasmid, which was electroporated into Δ*relA* containing pWRG99 (YZ313), generating strain YZ580. The primer pairs PYZ397/P71 and PYZ398/P69 were then used to amplify the two DNA fragments containing the T78I mutation, which were later fused via overlap PCR by using primers P69/P71. This DNA was electroporated into YZ580 to replace the SceI-Cam fragment and obtain the *spoT202*(T78I) strain. Similarly, for strain *spoT203*(R140C), primer pairs PYZ400/P71 and PYZ399/P69 were used to amplify the two DNA fragments containing the R140C mutation, which were fused together by using primers P69/P71. These DNAs were used to obtain the *spoT203*(R140C) strain. To make a clean in-frame deletion of *spoT*, oligo PYZ351 was used to replace the SceI-Cam fragment of strain YZ580. The respective *spoT* mutant sequences of the constructed strains were confirmed by PCR amplification and Sanger sequencing before use.

### Measurements of ppGpp by autoradiography

ppGpp were measured and quantitated the same as described in [24]. Two biological replicates were performed, and the average and SD were presented.

### MIC measurement

Overnight pre-cultures of the strains in MOPSr-Lp medium were washed with fresh MOPSr-Lp medium and inoculated (initial OD_600nm_=0.025, ca. CFU 2 x 10^^7^) into 200 μl fresh MOPSr-Lp medium supplemented with serially diluted antibiotics (starting concentrations of gentamycin 0.25 μg/ml, ofloxacin 0.125 μg/ml, ampicillin 25 μg/ml). The strains were allowed to grow in microtiter plates (LABSOLUTE, no. 7696794) for 72 hrs at 30°C with vigorous agitation (548 cpm, double orbit) in the Biotek Synergy H1 plate reader. Three biological replicates were performed.

### Selection of ppGpp^0^ suppressors in the presence of ampicillin

Strain YZ841 was grown in MOPSr-Lp medium supplemented with 12.5 μg/ml ampicillin for 72 hrs at 30°C with vigorous agitation (548 cpm, double orbit) in the Biotek Synergy H1 plate reader. Cells were washed with PBS to remove residual ampicillin before plating on an LB agar plate and incubated for 24 hrs at 37°C. Colonies (ca. 20) were picked and tested with their growth in MOPSr-Lp medium supplemented with 12.5 μg/ml ampicillin for 24 hrs, with the parental strain YZ841 as a negative control. Five candidate suppressors were selected that showed active growth within 24 hrs, as compared to the parental strain YZ841.

### Ampicillin killing assay

The killing assay was performed similarly as described [24]. Briefly, 16-hr overnight cultures of each strain were made in two ml MOPSr-Lp medium in a snap-tube (Sarstedt, no. 62.515.006). Approximately 10^^6^-2 x 10^^6^ cells of each strain were inoculated into 125 ml flasks containing 10 ml fresh MOPSr-Lp medium pre-warmed at 37°C. Cells were grown in a water bath at 37°C with agitation (160 rpm). After 2.5 hours of growth, lethal concentrations of ampicillin (250 µg/ml) were added in the cultures to kill the cells for 3 hrs or up to 5/8 hrs at 37°C with agitation (160 rpm). Right before antibiotic exposure, the total CFUs were determined by serial dilution, spotting, and incubation at 37°C for 24 hrs before counting. After each hour of antibiotic killing, 1.4 ml of cells were removed, spun down at 5000 rpm 3 min and then 14000 rpm 2 min. Cells were washed once with 1 ml of PBS, spun down as above, and finally resuspended in PBS. The CFUs of persister cells were determined by serial dilution, spotting and incubating on LB agar plate, and counting as above.

### Fluorescence microscopy

Bacterial cells out of overnight growth, regrowth in fresh MOPSr-Lp medium, and after different hours of ampicillin killing were visualized under a Nikon Eclipse Ti-E inverted microscope equipped with a 100x, 1.45 NA objective, a Zyla 5.5 sCMOS camera and a NIS-ELEMENTS software. All cells were washed with and resuspended in the M9Glc medium before imaging. Fiji and plugin MicrobeJ software [58] were used for automatic bacterial cell detection and quantification.

### Whole genome resequencing

Whole genome resequencing of the suppressor strains was performed at Genewiz and the sequence was analyzed and mutations identified using Artemis [59].

### Statistical analysis

GraphPad Prism v.8 was used throughout to analyze the data.

## SUPPLEMENTAL MATERIAL

Click here for supplemental data file.

All supplemental data for this article are available online at https://www.microbialcell.com/researcharticles/2023b-grucela-microbial-cell/.

## Abbreviations:

ppGpp: – guanosine penta and tetra phosphate,
MIC: – minimal inhibitory concentration,
PBP: – penicillin-binding protein,
RNAP: – RNA polymerase,
wt: – wild type.
