# Supplementary material for: Basal level of ppGpp coordinates Escherichia coli cell heterogeneity and ampicillin resistance and persistence
Source: Microb Cell. 2023 Oct 25;10(11):248–60. doi: 10.15698/mic2023.11.808 (PMC10625690; doi:10.15698/mic2023.11.808)
Supplement: Supplementary file 1 [file mic-10-248-s01.pdf]

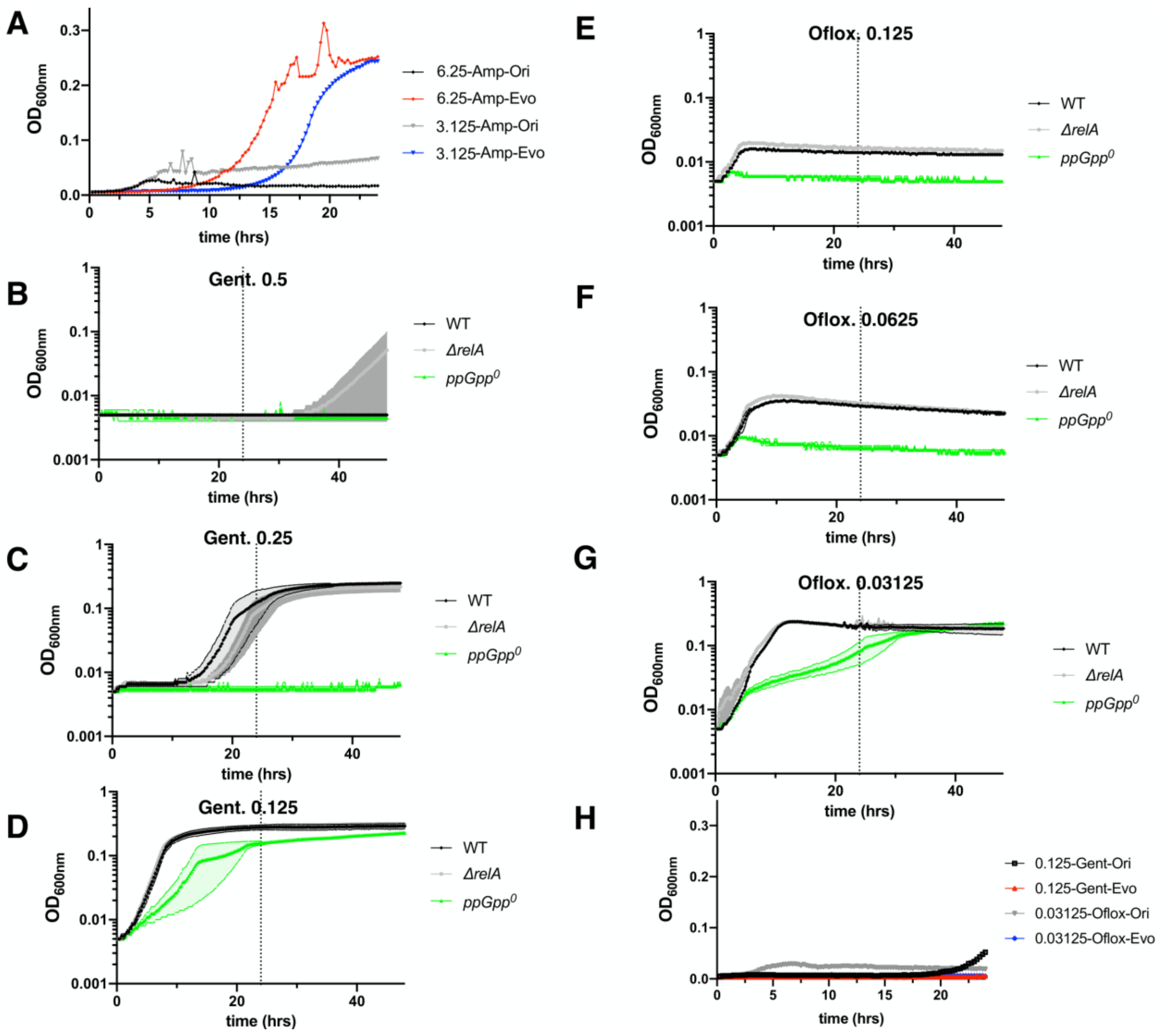

Supplemental Figure S1. Related to Figure 1. (**A**) The evolved (Evo) cells of the ppGpp^0 strain, after 72 hrs growth in the presence of ampicillin (both 6.25 and 3.125 µg/ml), grew up earlier than the original (Ori) ppGpp^0 strain. (**B, C, D**) Growth curves of the wt, *ΔrelA* and ppGpp^0 cells in the presence of gentamycin (0.5, 0.25, 0.125 µg/ml). (**E, F, G**) Growth curves of the wt, *ΔrelA* and ppGpp^0 cells in the presence of ofloxacin (0.125, 0.0625, 0.03125 µg/ml). Two biological replicates were performed, and the average and range were presented. (**H**) The evolved (Evo) cells of the ppGpp^0 strain, after 72 hrs growth in the presence of gentamycin and ofloxacin (0.125 and 0.03125 µg/ml, respectively), did not grow up earlier than the original (Ori) ppGpp^0 strain.

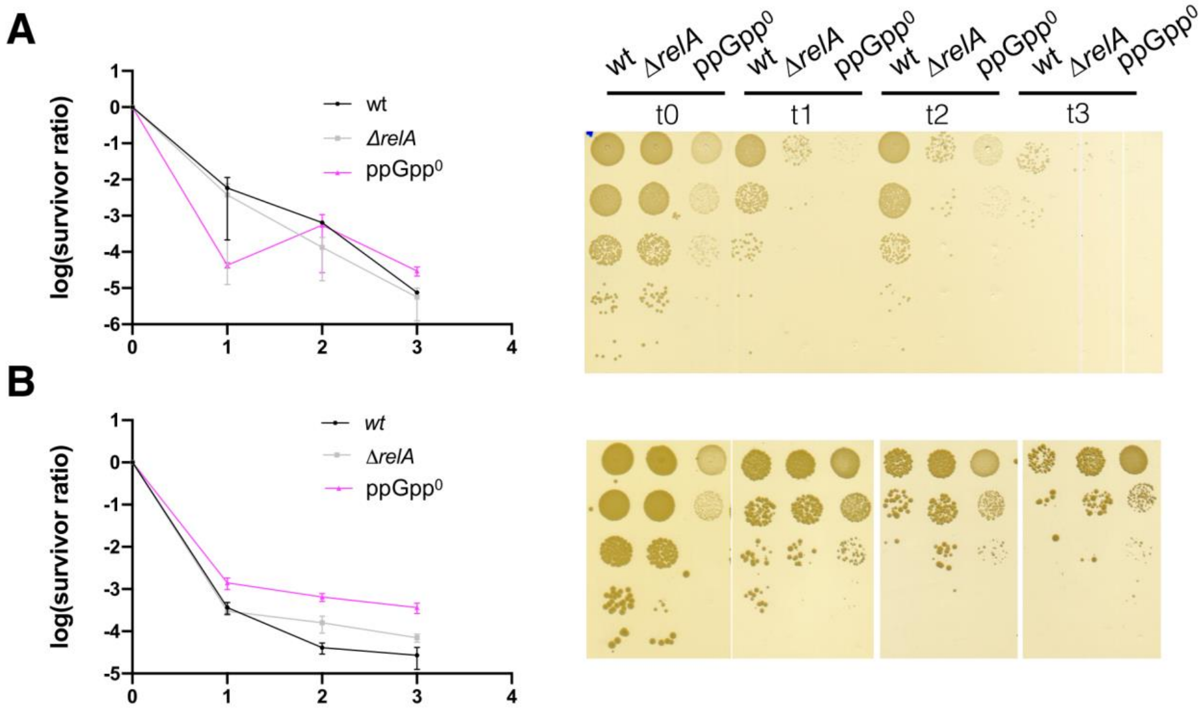

Supplemental Figure S2. (**A**) The gentamycin (2.5 µg/ml) and (**B**) ofloxacin (0.625 µg/ml) killing curves of the exponentially growing wt, *ΔrelA* and ppGpp^0 strains in MOPSr-Lp. Two (gentamycin) and six (ofloxacin) biological replicates were performed, and the averages and SDs are shown. Representative plates of the surviving cells after killing are shown to the right side of each panel.

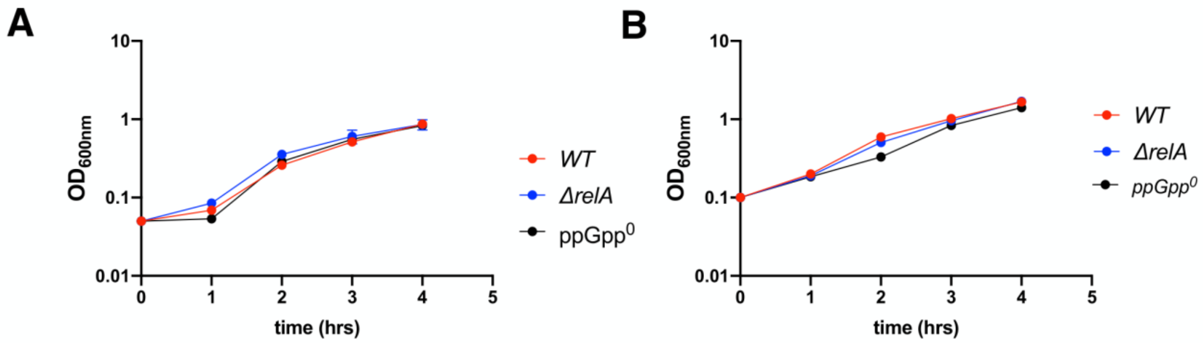

Supplemental Figure S3. Growth curves of the wt, Δ*relA* and ppGpp^0 cells in the MOPSr-Lp medium from initial OD_{600nm} of 0.05 (**A**) and 0.1 (**B**). Two biological replicates were performed, and the averages and SDs are shown.

Supplemental TABLE S1. Bacterial strains used in this study.

Strain	Relevant features	Reference
YZ142	MG1655, wild type *E. coli*	Laboratory stock
YZ38	MG1655 *ΔrelA*	[21]
YZ62	MG1655 *ΔrelA spoT207 ::cat*; CamR	[21]
YZ313	MG1655 *ΔrelA*, pWRG99	This study
YZ580	MG1655 *ΔrelA*, *spoT*(T78-E319)::IsceI-Cam; pWRG99	This study
YZ584	MG1655 *ΔrelA spoT203*(R140C)	This study
YZ585	MG1655 *ΔrelA spoT202*(T78I)	This study
YZ840	MG1655 *ΔrelA ΔspoT (colony-1)*	This study
YZ841	MG1655 *ΔrelA ΔspoT (colony-2)*	This study
YZ1094	MG1655 *ΔrelA ΔspoT rpoBD675A (colony-1)*	This study
YZ1097	MG1655 *ΔrelA ΔspoT rpoBD675A (colony-2)*	This study

Supplemental TABLE S2. Primers used in this study.

Number	Name	Sequence
pYZ69	DspoT-conf-F	gtcgtcgttaatcacaaagc
pYZ71	spoT-seq6-R	gcactgcataagcgaagtcg
pYZ351	80mer-ls(Spot)ifdel	ACAAAGCGGGTCGCCCttgtatctgtttgaaagcctgaatAAAGTCACCCGAAACCG AAATTAATGTTTTATGAACCCAA
pYZ395	60mer-spoTNt-ISceI-F	AACGCTGATGGCGGCGCTGCTGCATGACGTGATTGAAGATCTAGACTATATT ACCCTGTTATCCC
pYZ396	60mer-spoTNt-ISceI-R	TCTCCGCCATCTGGTCCATATCTTCGGTACGGATCTGGACATTTAAATGGCGC GCCTTAC
pYZ397	spoT-T78I-F2	GAAGATAtTCCCGCCACCTACCAG
pYZ398	spoT-T78I-R1	GGCGGGAaTATCTTCAATCACGTCATGCAG
pYZ399	spoT-R140C-R1	TGGGTACaGTCGGCAAGTTTGATGAGG
pYZ400	spoT-R140C-F2	TGCCGACtGTACCCACAACATGCGCA
